# Impact of maternal metabolic abnormalities in pregnancy on human milk and subsequent infant metabolic development: methodology and design

**DOI:** 10.1186/1471-2458-10-590

**Published:** 2010-10-06

**Authors:** Sylvia H Ley, Deborah L O'Connor, Ravi Retnakaran, Jill K Hamilton, Mathew Sermer, Bernard Zinman, Anthony J Hanley

**Affiliations:** 1 Department of Nutritional Sciences, University of Toronto, Toronto, Canada; 2 Physiology and Experimental Medicine, Department of Clinical Dietetics, Hospital for Sick Children, Toronto, Canada; 3 Division of Endocrinology, University of Toronto, Toronto, Canada; 4 Leadership Sinai Centre for Diabetes, Mount Sinai Hospital, Toronto, Canada; 5 Department of Pediatrics, Division of Endocrinology, Hospital for Sick Children, Toronto, Canada; 6 Obstetrics and Gynecology, Mount Sinai Hospital, Toronto, Canada; 7 Samuel Lunenfeld Research Institute, Mount Sinai Hospital, Toronto, Canada

## Abstract

**Background:**

Childhood obesity is on the rise and is a major risk factor for type 2 diabetes later in life. Recent evidence indicates that abnormalities that increase risk for diabetes may be initiated early in infancy. Since the offspring of women with diabetes have an increased long-term risk for obesity and type 2 diabetes, the impact of maternal metabolic abnormalities on early nutrition and infant metabolic trajectories is of considerable interest. Human breast milk, the preferred food during infancy, contains not only nutrients but also an array of bioactive substances including metabolic hormones. Nonetheless, only a few studies have reported concentrations of metabolic hormones in human milk specifically from women with metabolic abnormalities. We aim to investigate the impact of maternal metabolic abnormalities in pregnancy on human milk hormones and subsequently on infant development over the first year of life. The objective of this report is to present the methodology and design of this study.

**Methods/Design:**

The current investigation is a prospective study conducted within ongoing cohort studies of women and their offspring. Pregnant women attending outpatient obstetrics clinics in Toronto, Canada were recruited. Between April 2009 and July 2010, a total of 216 pregnant women underwent a baseline oral glucose tolerance test and provided medical and lifestyle history. Follow-up visits and telephone interviews are conducted and expected to be completed in October 2011. Upon delivery, infant birth anthropometry measurements and human breast milk samples are collected. At 3 and 12 months postpartum, mothers and infants are invited for follow-up assessments. Interim telephone interviews are conducted during the first year of offspring life to characterize infant feeding and supplementation behaviors.

**Discussion:**

An improved understanding of the link between maternal metabolic abnormalities in pregnancy and early infant nutrition may assist in the development of optimal prevention and intervention strategies and in the protection of nutritionally vulnerable offspring who are at risk for obesity and diabetes later in life.

## Background

Childhood obesity has emerged as an epidemic which is reflected in the rapidly increasing rates of youth onset type 2 diabetes [1-3]. Nutrition in infancy has been linked with lifetime effects on the pathogenesis of obesity, insulin resistance, and type 2 diabetes [4-6]. It has been proposed that nutrition signals during the early postnatal period may influence metabolic developmental pathways and, thereby, induce permanent changes to metabolic disease susceptibility [7,8]. Human breast milk is the most preferred food during this early postnatal period [9-11]. Several systemic reviews have reported that breastfeeding has a protective effect on obesity [4,5] and type 2 diabetes later in life [6]. The protective role of breastfeeding against childhood obesity and type 2 diabetes may be explained by a number of potential determinants including maternal metabolic abnormalities, infant feeding behaviors, and biological components of breast milk [12,13]. However, previously published large observational studies have not assessed whether maternal metabolic status in pregnancy and human milk components are responsible for the observed associations of breastfeeding with offspring obesity and type 2 diabetes.

Human milk contains not only nutrients but also an array of bioactive substances [14,15]. These human milk components function to transiently regulate tissue activities while the neonate's endocrine system matures [16]. Bioactive substances involved in metabolic regulation including adiponectin have been detected in human milk [17-19], and it has been hypothesized that these milk hormones may regulate growth and development in infancy and influence the programming of energy balance later in life [17]. Interestingly, one of these milk hormones, ghrelin, was reduced in the early postpartum among women who had gestational diabetes mellitus (GDM) [20]. In addition, mothers with diabetes often experience delayed onset of lactogenesis II, defined as the time to onset of copious milk secretion ≥72 hours postpartum [21]. Taken together, these reports suggest that the offspring of women with metabolic abnormalities may be exposed to compromised early nutrition and, therefore, are vulnerable to altered metabolic development and increased susceptibility to metabolic disease later in life.

Since offspring of women with obesity and diabetes are at increased long-term risk for type 2 diabetes [22,23], the impact of maternal metabolic abnormalities on early postnatal nutrition and infant metabolic trajectories is of considerable interest. Nonetheless, only a few studies have reported concentrations of metabolic hormones in human milk specifically from women with metabolic abnormalities in pregnancy [20]. None have investigated the associations of metabolic milk hormones in colostrum (early human milk before onset of lactogenesis II), from women with metabolic abnormalities, with early metabolic development in infancy.

### Maternal metabolic abnormalities and lactation

Women with obesity and/or diabetes often experience poor lactation performance [21,24-28]. High prepregnancy body mass index (BMI) was associated with delayed onset of lactogenesis II [29]. Delayed onset of lactogenesis II among overweight and obese women was explained by lower prolactin concentrations in response to suckling at 48 hours postpartum compared to normal-weight women [30]. Delayed lactogenesis II among overweight women in addition to excessive gestational weight gain have been associated with the early cessation of breastfeeding [27,31,32]. Studies which investigated milk composition of women with type 1 diabetes reported that the early milk samples had altered lactose, citrate, and total nitrogen concentrations [33,34]. However, these studies were limited by small sample sizes which precluded adjustment for potential confounding variables including delivery complications, maternal behavior, and socioeconomic status (SES). In addition, these studies did not investigate concentrations of milk hormones that might be involved in metabolic regulation.

### Maternal metabolic abnormalities, breastfeeding and childhood obesity

Although maternal metabolic abnormalities have been documented to affect offspring metabolic status [22], only a few studies have examined the impact of neonatal feeding practices by women with metabolic abnormalities on subsequent offspring obesity or diabetes [35,36]. In a study from Germany, infants were provided with human milk from their mothers with diabetes or donor human milk from women with normal glucose tolerance [35]. Children who consumed a higher amount of human milk from mothers with diabetes during the first week of life were more likely to have a higher % relative body weight at 2 years of age [35]. Neither ingestion of human milk from women with diabetes during the first 2 to 4 weeks of life nor the duration of breastfeeding had an independent influence on risk of childhood overweight after adjusting for neonatal feeding practice during the first week of life [36]. The authors, therefore, concluded that the first week of life was a critical window for nutritional programming of offspring who ingested milk from mothers with diabetes. Human milk composition during this first week of life, however, was not reported in this study. Therefore, it is unknown which components in human milk contributed to this difference.

### Metabolic hormones in human breast milk

In addition to nutrients, human milk contains bioactive substances including metabolic hormones [14]. A number of metabolic hormones, including leptin, adiponectin, and ghrelin [15,18,19,37-40], have been detected in human milk. In serum, these hormones regulate energy balance [41,42] and obesity-induced inflammatory signaling pathways that contribute to type 2 diabetes [43].

**Figure 1:**
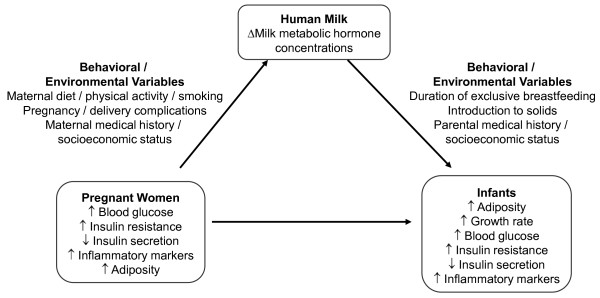
**Conceptual model**.

Although several reports have been published on human milk leptin [15,37,44,45], limited information is available on whether milk leptin concentrations vary from women across a spectrum of metabolic abnormalities in pregnancy, ranging from normal to obese/insulin resistant to the full range of glucose tolerance in pregnancy. Previous population-based longitudinal reports on the association of milk adiponectin with offspring anthropometric measures did not provide detailed metabolic characteristics of mothers nor did they study milk composition specifically in colostrum [46,47]. One study reported that ghrelin concentration in colostrum was reduced among women who had GDM, but the difference was normalized and not observed in mature milk [20]. This study, however, was conducted in a small sample size and therefore the observation needs to be confirmed with a larger sample size. In addition, women who are obese and/or have diabetes are known to experience delayed onset of lactogenesis II [21,27]. Hence, offspring of mothers with metabolic abnormalities may be exposed to compromised early nutrition due to a lack of accessibility to human milk and/or to an altered composition of human milk (Figure 1).

With emerging evidence indicating that metabolic hormones are present in human milk, the impact of milk hormones on the early metabolic regulation and potential programming of energy balance requires an improved understanding. Since adiponectin and leptin receptors are present in the human gastrointestinal tract [48-51], it is conceivable that milk hormones may have a functional role locally via receptors in the stomach and intestine. The immaturity of the neonatal gut barrier may also allow for absorption and survival of various hormones from human milk, which would allow milk hormones to systemically influence metabolic development of the infant. Goldman [52,53] and Koldovsky [54] have reviewed characteristics of the neonatal gastrointestinal tract that would allow for survival of human milk components in this setting, including delayed production of pancreatic proteases and gastric acid, the presence of antiproteases and inhibitors in human milk, and the possible existence of higher permeability in the neonatal gut to macromolecules. Disordered hormone concentrations in human milk during a critical period may lead to sub-optimal development of fundamental regulatory systems in offspring [8]. For example, neural projection pathways from the arcuate nucleus of the hypothalamus are permanently disrupted in leptin-deficient mice [55]. Among these mice, leptin treatment in adulthood did not reverse the defects, whereas the treatment in neonates preserved the development of these pathways [55]. Optimal milk hormone levels may promote the normal development of hypothalamic pathways influencing energy homeostasis throughout life. Only a few studies have reported concentrations of metabolic hormones in human milk specifically from women with metabolic abnormalities in pregnancy [20]. None have investigated the associations of metabolic milk hormones in colostrum, from women with metabolic abnormalities, with early metabolic development in infancy. We aim to address these knowledge gaps within the context of our ongoing mother and infant cohort studies. This study is conducted within a hospital setting which allows detailed characterization of clinical events during pregnancy and delivery in addition to lifestyle information collected throughout late pregnancy and the first year postpartum through scheduled contacts with participants. The purpose of this report is to present the methodology and design of the study investigating the impact of maternal metabolic abnormalities in pregnancy on human milk hormones and subsequently on infant development over the first year of life.

### Study objectives

Our overall aim is to investigate the impact of maternal metabolic status in pregnancy on breastfeeding, human milk composition, and infant development. The main specific objectives are as follows:

1. To investigate concentrations of metabolic hormones in colostrum of lactating women who had varying metabolic characteristics in pregnancy (i.e. a range of adiposity, subclinical inflammation, insulin resistance, beta-cell dysfunction, and glucose tolerance);

2. To compare metabolic hormone concentrations in colostrum with concentrations in mature breast milk at 3 months postpartum;

3. To study the associations of milk hormones in colostrum from mothers with varying metabolic characteristics with infant metabolic characteristics (i.e. adiposity and growth) over the first year of life; and

4. To investigate the associations of maternal metabolic abnormalities with delayed onset of lactogenesis II, defined as onset of copious milk secretion ≥72 hours postpartum.

## Methods/Design

### Study setting and design

The current study is conducted within ongoing mother and infant prospective cohort studies investigating early events in the natural history of type 2 diabetes and vascular disease. These studies are being conducted at Mount Sinai Hospital and the Hospital for Sick Children in Toronto, Ontario, Canada [56-59]. The current study protocol was approved by the Mount Sinai Hospital Research Ethics Board.

**Figure 2:**
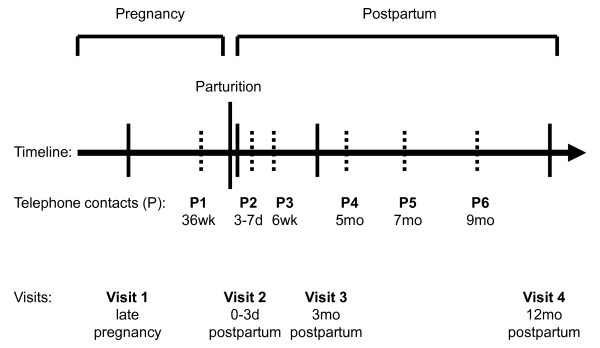
**Schedule of four assessment visits and interim telephone calls from late pregnancy to 1-year postpartum**.

Standard obstetrical practice in Canada involves universal screening for GDM in all pregnant women at 24-28 weeks gestation by a glucose challenge test (GCT), wherein plasma glucose concentration is measured 1 hour after ingestion of a 50 g glucose load [23]. If the plasma glucose concentration is ≥ 7.8 mmol/L, the patient is referred for a diagnostic oral glucose tolerance test (OGTT), in which plasma glucose values are measured while fasting and then hourly for 3 hours following ingestion of 100 g of glucose. In clinical practice, the OGTT would typically only be ordered if the GCT were abnormal, whereas, in this study, the baseline pregnancy OGTT was completed in all participating women. In addition to the baseline OGTT visit, women were asked to participate in three follow-up visits and six telephone contacts from late pregnancy to one year postpartum (Figures 2).

### Study participants and recruitment

Pregnant women were recruited in outpatient clinic waiting areas at Mount Sinai Hospital in Toronto, Canada, a large tertiary care centre where over 7000 pregnancies per year are followed. The inclusion criteria were singleton or twin pregnancy, aged 20 years or older at the time of recruitment, and intention to breastfeed. Women who reported to have pre-existing diabetes were excluded. Written informed consents were collected from 271 pregnant women. A total of 55 participants were withdrawn from the study without completing the baseline visit. Forty seven of 55 were unable to visit the study testing unit during late pregnancy due to personal reasons including difficulty taking time off from work in addition to the time required for routine clinical obstetric visits. Eight of 55 women initiated the baseline visit but were unable to complete the 3-hour OGTT due to clinical reasons including nausea and vomiting. A total of 216 participants completed the baseline pregnancy characterization between April 2009 and July 2010.

### Sample size justification

Sample size calculations were performed using PASS (Kaysville, UT). We estimated that baseline pregnancy characterization of between 210 and 220 women was required to address our four main specific objectives based on the following estimations.

To assess variation in metabolic hormones in colostrum from women with varying pregnancy metabolic characterization (objective 1), a sample size of 82 was required to provide 80% power to detect a slope of 0.10 at the significance level of 0.05; this sample size would allow detection of the correlation of 0.30. Based on previous literature, we expected that some women, especially overweight/obese women might experience delayed onset of lactogenesis II [26,29,31,60], and therefore might have difficulty expressing colostrum samples. We considered that 50% of our participating women would be in overweight/obese pre-pregnancy BMI categories (i.e. BMI > 25 kg/m^2^) [56] and that 20% of normal weight women and 35% of overweight/obese women would experience delayed lactogenesis II [26,29,31,60]. After accounting for 15% loss to follow-up by the visit 2, a sample size of 135 was required to assess objective 1.

To test variation in metabolic hormone concentrations in colostrum and mature milk (objective 2), we considered that 25% of lactating women might discontinue providing human milk before 3 month postpartum based on previous local and national data [61,62]. After accounting for additional 10% loss to follow-up from visit 2 to 3, a sample size of 199 was required to assess objective 2.

To assess the impact of milk hormone concentrations on infant metabolic characteristics over the first year of life (objective 3), we accounted for additional 5% loss to follow-up from visit 3 to 4. This provided a sample size requirement of 210 women.

To study delayed onset of lactogenesis II from women with varying metabolic characteristics (objective 4), we aimed to complete pregnancy characterization for a sample that would provide 80% power at a 0.05 significance level to detect a difference in the outcome of delayed onset of lactogenesis II when the independent variable (e.g. BMI) was increased to one standard deviation above the mean. This change corresponded to an odds ratio of 1.8 in a logistic regression model with adjustment for other independent variables that themselves account for an R-Squared of 0.3 (or 30%). After considering a possible 20-30% overall loss to follow-up/missing data rate from baseline to the first week postpartum interview, we estimated that a sample size of 193-220 women was required to test objective 4.

### Characterization of maternal metabolic status and data collection

Data collection for this prospective cohort study occurs during four study visits and six telephone contacts from late pregnancy to one year postpartum (Figure 2). Follow-up visits and interim telephone interviews are expected to be completed in October 2011.

#### Visit 1 (baseline): late second and early third trimester of pregnancy

Participating women underwent a 3-hour 100 g OGTT in late pregnancy during which blood samples were drawn at fasting and at 30, 60, 120 and 180 minutes post-load. The OGTT on participating women in this study provides four categories of maternal glucose tolerance in pregnancy [56]:

a. women with GDM defined as two or more of the following [63]: fasting glucose ≥5.8 mmol/L, 1-hour blood glucose ≥10.6 mmol/L, 2-hour blood glucose ≥9.2 mmol/L, or 3-hour blood glucose ≥8.1 mmol/L;

b. women with an OGTT indicating gestational impaired glucose tolerance defined by exceeding only one of the above glycemic thresholds [56];

c. women with an abnormal GCT but a normal OGTT (i.e. none of the above criteria); and

d. women with a normal GCT and a normal OGTT.

Interviewers administered questionnaires to obtain information on demographic, medical history, smoking behavior, and physical activity using the Baecke instrument [56,57]. Dietary intake was collected using a modified Block food frequency questionnaire (FFQ) in which women were asked to recall usual intake during the second trimester of pregnancy [64,65]. The Block FFQ has been validated in several populations [66,67], including women in Ontario, Canada [68]. The modified Block FFQ focuses on intake during the previous 3-month time period and has been validated within a pregnant population [69,70]. The questionnaire is designed to take approximately 30 minutes to complete and is self-administered. Women's height and weight were measured following standardized anthropometric protocols [71].

#### Telephone contact: 36 weeks gestation

Participating women are contacted at 36 weeks gestation via a telephone call. Participants are instructed to complete and return a modified Block FFQ recalling their usual dietary intake during the third trimester of pregnancy. Participants were provided with a FFQ in a postage-paid return-addressed envelop during the baseline visit for this purpose.

#### Visit 2: the first 3 days postpartum

Participating women and their neonates are visited in the hospital within the first 3 days postpartum. Infant anthropometry measurements are performed following standardized procedures (NHANES III Anthropometric Procedures) [72]. For length, infants are placed supine on a measuring device, with a fixed headboard and a moveable footboard (Seca, Hamburg, Germany), and measured to the nearest 0.1 cm. Infants are weighed supine using an electronic infant scale (Medela BabyWeight; Medela Inc, Mississauga, Canada). For head and abdominal circumference, a non-stretchable, plasticized measuring tape is used to measure to the nearest 0.1 cm. The tape is wrapped around the head of the infant superior to the eyebrows and ears to assess the largest head circumference. For abdomen circumference, the tape is wrapped around the abdomen above umbilicus with the infant in the supine position, and the expiration circumference is recorded. Three measurements are performed for each. Delivery and birth medical information of mothers and infants are obtained from a hospital clinical database.

Colostrum samples are collected by hand expression or an electric breastpump (Medela Electric Double Breastpump; Medela Inc, Mississauga, Canada), whichever is preferred by a participant, during their hospital stay. If the participating woman is unable to express milk during the hospital stay, a home visit is conducted by study personnel. Colostrum or an early human milk donation is accepted up until day 7 of infant life (first 24 hours is considered as day 0).

#### Telephone interview: the first week of postpartum

On day 3 postpartum, mothers are contacted via a telephone call (or visited in the hospital) to obtain information on the timing of onset of lactogenesis II and early postpartum infant feeding and supplementation behaviors. Mothers are asked to recall the nearest hour when they started to feel their "milk-coming-in". Specifically, mothers are asked to recall the presence of a "tingling" feeling in the breast, fullness, and swelling. This technique for assessing onset of lactogenesis II by maternal perception has been validated with test weighing [73]. If the mother has not experienced lactogenesis II by day 3 postpartum, the women is contacted again on day 7 postpartum (± 2 days). If the woman reports no "milk-coming-in" by day 7, the time to onset of lactogenesis II is recorded as greater than 7 days postpartum.

#### Telephone interview: 6 weeks postpartum

Mothers are contacted at 6 weeks postpartum to obtain information on infant feeding, specifically, exclusivity and duration of breastfeeding and supplementation behaviors. Mothers are asked to recall dates of introduction to infant formula, cow's milk, and other fluid and solids, if the event has taken place. If the mother reports to have discontinued breastfeeding, the last breastfeeding date and reasons for discontinuation are collected.

#### Visit 3: 3 months postpartum

Mothers and infants are invited to our research study unit for a follow-up assessment at 3 months postpartum (± 1 month). Mothers are asked to perform complete breast expression using a double electric breastpump. Mature human milk samples are collected for analysis. Mothers complete a 2-hour 75 g OGTT. Interviewers administer questionnaires asking about parental SES status, paternal medical history, maternal postpartum physical activity/smoking, and infant feeding behaviors. A self-administered modified 3-month Block FFQ is completed asking mothers to recall their food intake during the first 3 months postpartum.

Infant anthropometric assessments are performed using standardized anthropometric procedures (NHANES III Anthropometric Procedures) [72], as described for visit 2. In addition, skinfold thickness measurements are performed using a skinfold caliper (Harpenden skinfold caliper; John Bull British Indicators Ltd, West Sussex, United Kingdom). Triceps, biceps, suprailiac, and subscapular skinfold thickness are measured on the right side of the body under standard conditions [74]. Validation studies have shown that skinfold thickness measurements correlate with body fat assessed by direct measurements including dual-energy x-ray absorptiometry in infants [75] and children [76]. Three measurements are performed for each anthropometric variable.

#### Telephone interviews: 5, 7 and 9 months postpartum

Mothers are contacted at 5, 7 and 9 months postpartum via a telephone call and asked to recall infant feeding and supplementation behaviors as described for the 6 week phone interview.

#### Visit 4: 12 months postpartum

Mothers and infants are invited to the research study unit for a follow-up assessment at 12 months postpartum. Mothers complete a 2-hour 75 g OGTT. Infant anthropometric assessments are performed using standardized anthropometric protocols as described for the 3 month postpartum assessments. Infants are weighed using an electronic infant scale appropriate for infants at 1-year (Seca, Hamburg, Germany). Infant feeding questionnaires are administered asking mothers to recall infant feeding and supplementation behaviors.

### Biochemical Procedures and Analyses

Maternal serum samples are processed and analyzed for glucose and insulin concentrations according to established protocols at the Banting and Best Diabetes Centre laboratory, Mount Sinai Hospital, Toronto, Ontario, Canada. Aliquots of serum/plasma are prepared and frozen immediately at -80°C for additional assays (e.g. inflammatory markers, adipokines, and lipids). Insulin sensitivity and beta-cell function are assessed by validated indices derived from insulin and glucose measurements during the OGTT [77,78].

For human milk samples, whole milk samples are divided into aliquots and stored at -80°C until analysis. Before performing assays to measure metabolic hormone concentrations, milk samples are sonicated to disrupt the membrane vesicles and to allow immunodetection of milk hormones because adipokines may be present in milk fat globules [79]. Skim milk is prepared by centrifugation to separate milk fat from the liquid phase.

### Statistical Analyses

Distributions of continuous variables will be assessed for normality, and transformations of skewed variables will be used in statistical analyses as appropriate. Descriptive statistics for continuous variables will be summarized as mean ± standard deviation or median (25^th^-75^th ^percentile) for variables with a skewed distribution. Categorical variables will be summarized using proportions. Welch's modified t test or Chi-Square test will be performed for continuous and categorical pairwise group comparisons as appropriate. Bivariate associations of continuous variables will be assessed using Spearman correlation analysis.

The following statistical analyses will be performed to address the main objectives. Multiple linear regression will be performed to assess study objectives 1, 2, and 3 with adjustment for potential covariates including maternal lifestyle, SES, and medical history. The main outcome and exposure variables and covariates for these models, by objective, are presented in detail in Table 1. For objective 1, the outcome variable will be metabolic hormone concentrations in colostrum, while the exposure variables will be maternal pregnancy metabolic characteristics. For objective 2, the outcome variable will be concentrations of metabolic hormones in mature breast milk at 3 months postpartum, while the exposure variables will be concentrations of metabolic hormones in colostrum adjusting for covariates including maternal metabolic characteristics at 3 months postpartum. For objective 3, the outcome variable will be metabolic characteristics of offspring during the first year of life, while the main exposure variables will be concentrations of metabolic hormones in colostrum adjusting for potential covariates including maternal gestational metabolic characteristics. This covariate adjustment will enable us to assess the associations between milk hormones and offspring metabolic characteristics independent of maternal gestational metabolic variables. Other potential confounders to be evaluated in these models are listed in Table 1. For study objective 4, multiple logistic regression will be performed with potential adjustment for covariates including maternal lifestyle, SES, and medical history. In these models, the outcome variable will be defined as the presence of delayed lactogenesis II; the main exposures will be maternal metabolic abnormalities (i.e. obesity, hyperglycemia, insulin resistance) (Table 1).

**Table 1:** Summary of statistical analysis plans

Objective	Statistical technique	Outcome variable	Main exposures	Covariates
**1**	Linear regression	concentrations of metabolic hormones in colostrum (e.g. adiponectin)	maternal metabolic characteristics (e.g., glucose intolerance, insulin resistance, obesity)	maternal lifestyle, SES, medical history
**2**	Linear regression	concentrations of metabolic hormones in mature breast milk at 3 months postpartum	concentrations of metabolic hormones in colostrum	maternal lifestyle, SES, medical history, maternal metabolic characteristics
**3**	Linear regression	metabolic characteristics in offspring (e.g. infant growth, adiposity) during the first year of life	concentrations of metabolic hormones in colostrum	maternal lifestyle, SES, medical history, maternal metabolic characteristics, infant feeding/supplementation behaviors
**4**	Logistic regression	delayed onset of lactogenesis II	maternal metabolic characteristics (e.g., glucose intolerance, insulin resistance, obesity)	maternal lifestyle, SES, medical history

Abbreviations: SES, socioeconomic status

## Discussion

We anticipate that this study will allow us to investigate the impact of maternal metabolic characteristics in pregnancy on breastfeeding and infant development. An improved understanding of maternal metabolic abnormalities and infant nutrition may assist in developing prevention and intervention strategies which will protect vulnerable offspring exposed to suboptimal nutrition.

The main challenge of this study is that we have a narrow biological window of time to collect colostrum samples. We, therefore, have developed a computerized notification protocol to alert the study personnel when participants are admitted to the hospital maternity unit and delivered their infants. However, it is expected that some women may experience difficulty expressing and donating milk samples during the hospital stay. We have considered this aspect into our sample size power calculation. In addition, we have also developed an additional step within our protocol to visit mothers at home to increase the opportunity to collect early human milk samples within the first week postpartum. The strength of the study is that it will yield novel insights into the impact of maternal metabolic abnormalities on human milk hormones and subsequently on the offspring's early metabolic trajectory. This study may assist in developing optimal strategies to support the development of nutritionally vulnerable offspring exposed to maternal metabolic abnormalities and, therefore, reducing risk for obesity, type 2 diabetes, and vascular disease later in life.

## Competing interests

The authors declare that they have no competing interests.

## Authors' contributions

All authors contributed to the conception and/or design of the study. SHL drafted the manuscript, and all of authors read and approved the final manuscript and contributed in revising the manuscript critically for important intellectual content.

## Pre-publication history

The pre-publication history for this paper can be accessed here:

http://www.biomedcentral.com/1471-2458/10/590/prepub
